# Primary psoas abscess due to Streptococcus milleri

**DOI:** 10.1186/1476-0711-7-7

**Published:** 2008-02-26

**Authors:** Nitin B Bagul, Abeywardana MS Abeysekara, Sabu Jacob

**Affiliations:** 1 Department of Surgery, University Hospital of North Tees, Stockton, UK; 2 Department of Surgery, King George Hospital, Goodmayes, Essex, UK

## Abstract

Primary Psoas abscess (PPA) is an infrequent clinical entity with obscure pathogenesis and vague clinical presentation. High index of clinical suspicion is required for the diagnosis of psoas abscess. We also emphasises the importance of bacteriological confirmation of microorganism involved, although *Staphylococcus aureus *remains the commonest pathogen. We report an extremely rare case of PPA caused by *Streptococcus milleri*. Only one case has been reported in literature so far.

## Introduction

Primary psoas abscess (PPA) is extremely rare clinical entity, which potentially carries high mortality and morbidity if diagnosis is delayed. A PPA has no obvious focus of infection, with the commonest pathogen being *Staphylococcus aureus *(SA). We report an extremely rare case of PPA caused by *Streptococcus milleri *(SM). Only one case has been reported in literature as far as our knowledge [1]. It carries a good prognosis provided early drainage is performed and parenteral antibiotic therapy is administered to patient. High index of clinical suspicion is required for the diagnosis of psoas abscess (PA). We also emphasises the importance of bacteriological confirmation of microorganism involved.

## Illustrative case history

**Figure 1:**
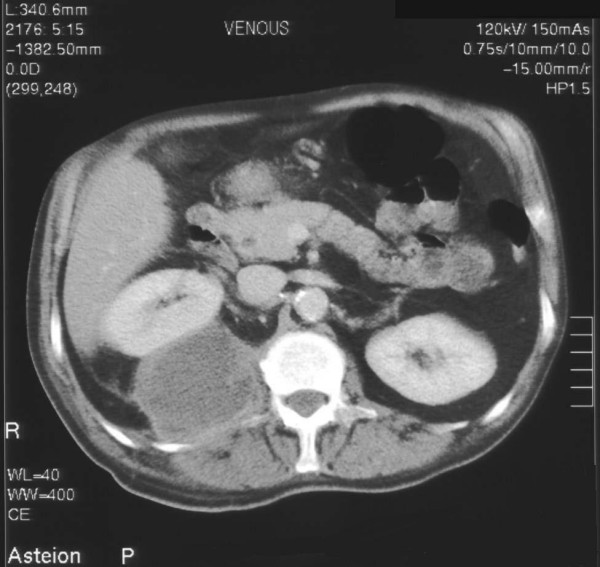
Computed tomogram of abdomen showing right psoas abscess abutting the right kidney.

**Figure 2:**
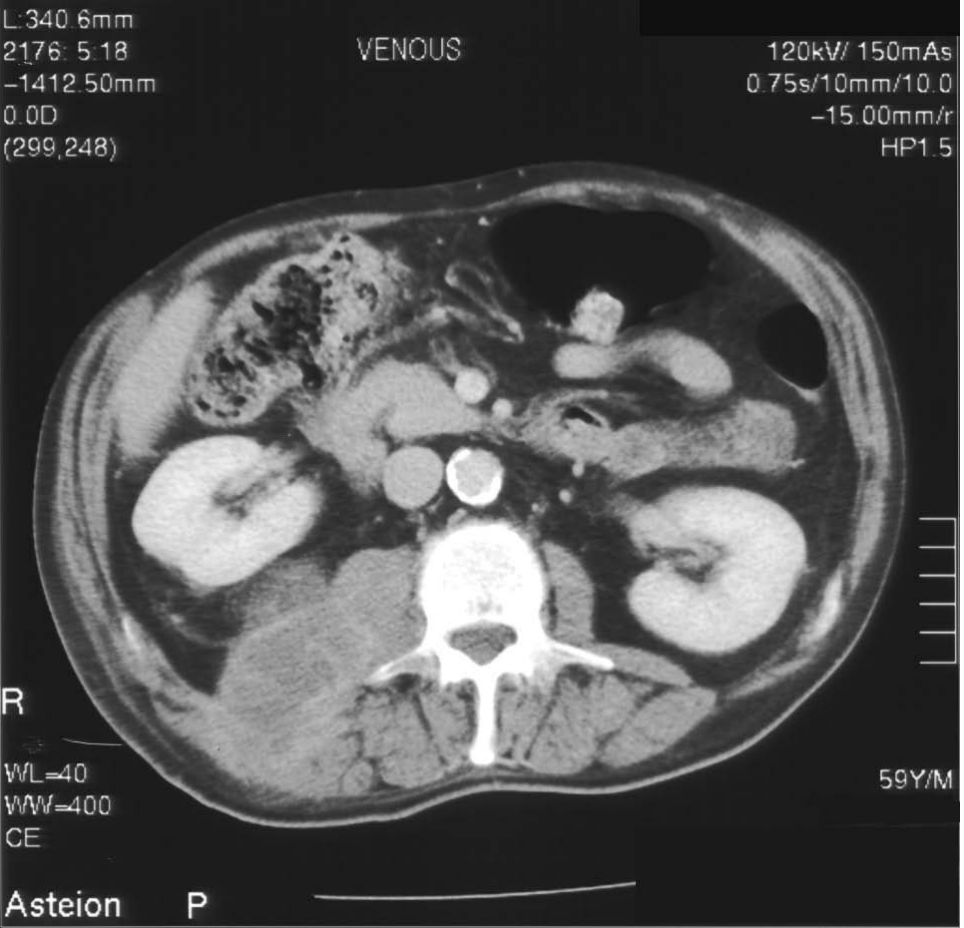
Computed tomogram of abdomen showing right psoas abscess extending from above the right kidney.

A 57-year-old man was admitted to hospital with seven-day history of right upper quadrant and loin pain radiating to right leg and umbilicus. Patient also complained of fever, chills, general malaise, decreased appetite. Patient denied any urinary symptoms. Patient had significant past medical history of coronary artery bypass surgery 5 years ago and laparoscopic cholecystectomy 2 years ago. On general examination he was pale, temp-37.5 degree Celsius. On physical examination he had fullness in right flank, which was tender and fluctuant. Laboratory investigation revealed leucocytosis of 14.9 × 10^9^/l. Plain abdominal radiograph showed a soft tissue shadow on right upper quadrant. Ultrasonography (USS) guided aspiration was undertaken and 250 ml of thick brown blood stained pus was drained and sent for microscopy and culture. Drain was kept in situ. Microbiology reported SM as causative organism sensitive to penicillin and erythromycin. Patient was started on intravenous 1.8 g qds benzyl penicillin for 4 weeks. Computed Tomography (CT) and barium enema was performed to rule out bowel origin (Figure 1 &2). Magnetic resonance imaging scan was performed which showed abscess in posterior abdominal wall extending to retro peritoneum abutting the right kidney. Drain was removed in 20 days after ultrasound confirmation. The patient's general condition improved over a period of 28 days during the stay in hospital. Patient was subsequently discharge and on follow up of one year he is doing fine.

## Discussion

Mynter first described psoas abscess in 1881 refereeing as psoitis [2]. PA is a relatively uncommon condition in western countries. PA may be classified as primary or secondary, depending on the presence or absence of underlying infection. PPA occurs probably as a result of haematogenous spread of an infectious process from an occult source in the body. Incidence of PA was 12 cases per year worldwide in 1992 [3]. This was a significant increase from the calculated occurrence of 3.9 cases per year before 1985 [4]. The increase was attributed to improved diagnosis with the widespread use of computed tomography. PPA is most prevalent in young patients and occurs rarely in the elderly population. Certain groups of immunocompromised patients, such as diabetics, the elderly, patients on steroids, patients with malignancies, and alcoholics, usually present with infrequent sites of infection. SA is the causative organism in over 88% of patients with PPA [4], *Streptococcus species *4.9% and *Escherichia coli *2.8%. Other rare pathogens reported are *Mycobacterium tuberculosis, Streptococcus species, Escherichia coli, Salmonella enteritidis, Pneumococcal, Pseudomonas aeruginosa, Proteus Mirabilis, Yersinia enterocolitica, Bacteroides, Pasteurella multocida, Klebsiella, Serratia marcescens Mycobacterium kansasii, and Mycobacterium xenopi. Methillicin resistant Staphylococcus aureus *is also known pathogen [5].

SM is very rare organism to cause PA. It is Gram positive coccus which is usually involved in endocarditis, peritonitis, intracranial abscess, intraspinal abscess, liver abscess and lung abscess [6]. It belongs to *Streptococcus milleri group *along with *Streptococcus intermedius *and *anginosus*.

The classical symptoms of patients with PPA are fever, flank or abdominal pain, and limp. The patient mentioned in our case report presented with similar symptoms. Other symptoms may include malaise, weight loss, or presentation with a mass. Physical findings, such as external rotation of the ipsilateral hip, flank tenderness, and fullness of the flank may be seen. Laboratory tests may reveal raised white cell count, anaemia, and elevated erythrocyte sedimentation rate, and bacteriological confirmation of microorganism is utmost importance.

Radiographic imaging studies help in diagnosis and may also help in finding an underlying cause. Plain abdominal x-ray may reveal an abnormal psoas shadow or a soft tissue mass. USS of the abdomen may demonstrate a hypoechoic mass suggestive of PA, but cannot identify the cause of the abscess. CT scan of the abdomen with contrast is the most efficient and accurate imaging study in diagnosing a PA. CT scanning is now used as the first line of investigation. CT scan of the abdomen not only helps in diagnosis, but also in identification of the etiology, for therapeutic purposes, and postoperative follow-up [7,8].

An adequate knowledge of the causative organisms should guide the initial choice of antibiotics. But at the same time the importance of bacteriological confirmation of micro organism involved should not be ignored. It has been suggested that in PPA, antistaphylococcal antibiotic therapy should be started before final bacteriologic diagnosis [4]. However, the identification of non-staphylococcus organisms in some patients with PPA and the identification of staphylococcus in patients with secondary PA, makes it prudent in all cases of psoas abscess to start treatment with broad spectrum antibiotics like cephalosporins, quinolones, imipenem and clindamycin pending final bacteriologic diagnosis [9]. Antibiotics are sometimes continued up to four to six weeks after complete drainage of the abscess.

Drainage can be surgical or radiologically. Percutaneous drainage may be difficult in some patients because of the location of the abscess, but whenever possible it should be employed. Even in patients with complex, multiloculated abscesses, percutaneous drainage should be attempted and open surgical drainage should be reserved if percutaneous drainage fails. Patients with secondary psoas abscess require correction of their underlying disease in addition to the drainage procedure. Extraperitoneal drainage is a safe, effective method of draining these abscesses. Compared to percutaneous drainage, an advantage with open drainage is that debridement of adjacent tissues can be done, which may help in shortening recovery time [10]. The prognosis of PPA is better compared to secondary abscess [4].

## Conclusion

High index of clinical suspicion is required for the diagnosis of psoas abscess. We also emphasises the importance of bacteriological confirmation of microorganism involved, although *Staphylococcus aureus *remains the commonest pathogen.

## Competing interests

The author(s) declare that they have no competing interests.

## Authors' contributions

All authors were involved in the care of the patient. NBB drafted the manuscript. AMSA, SJ critically reviewed and improved the manuscript. All authors read and approved the final manuscript.

## Consent

Written informed consent was obtained from the patient for participation in this research.

A copy of the written consent is available for review by the Editor-in-Chief of this journal.
